# Combined Glycoprotein Mutations in Rabies Virus Promote Astrocyte Tropism and Protective CNS Immunity in Mice

**DOI:** 10.3390/v18020181

**Published:** 2026-01-29

**Authors:** Mirjam Anna Rita Bertoune, Corinna Kolbe, Ann-Cathrin Werner, Maren Steinmetz, Bernhard Dietzschold, Eberhard Weihe

**Affiliations:** 1 Department of Medical Cell Biology, Institute of Anatomy & Cell Biology, Marburg University, 35037 Marburg, Germany; 2 Institute of Anatomy & Cell Biology, Marburg University, 35037 Marburg, Germany; 3 Department of Microbiology and Immunology, Thomas Jefferson University, Philadelphia, PA 19107, USA

**Keywords:** rabies virus, tropism, astrocytes, antiviral immune response

## Abstract

Rabies virus (RABV) causes fatal encephalitis once it invades the central nervous system (CNS), and treatment options are extremely limited at this stage. We investigated the recombinant RABV variants SPBN, SPBNGA (glycoprotein substitution R333E), SPBNGAK (R333E plus N194K), SPBNGAS (R333E plus N194S), and TriGAS (three copies of the R333E/N194S glycoprotein). We evaluated their cellular tropism and immune activation in an intracerebral mouse infection model using immunohistochemistry and confocal immunofluorescence. SPBNGAK (R333E/N194K) resulted in mixed neuronal and astrocytic infection and lethal disease. In contrast, the R333E/N194S mutations in the GAS variants were associated with reduced neuronal infection and apparent astrocyte-restricted infection patterns. This tropism shift coincided with microglial activation (allograft inflammatory factor 1, amoeboid transformation) and astrocytic activation (nestin), along with T-cell infiltration and endothelial activation that persisted beyond viral clearance. SPBNGAK-infected astrocytes expressed nestin, while GAS variant-infected astrocytes remained nestin-negative and were rapidly cleared. Intracerebral co-inoculation of astrocytotropic TriGAS with the lethal neurotropic DOG4 strain was associated with survival and a marked reduction in detectable DOG4 neuronal infection. These findings suggest that glycoprotein-mediated astrocyte tropism may be associated with altered immune responses after rabies CNS invasion. While mechanistic causality cannot be inferred, these observations may inform the design of future studies exploring astrocyte-restricted RABV infection in therapeutic-related contexts.

## 1. Introduction

Rabies virus (RABV) infects mammals and causes fatal encephalitis. Death can be prevented only by preexposure vaccination with inactivated RABV or by immediate postexposure prophylaxis with vaccines and immunoglobulins, provided treatment is initiated before central nervous system (CNS) invasion. These interventions are costly and logistically challenging, particularly in endemic areas. Combined with the absence of a cure for clinical rabies [1,2,3], this contributes to an estimated 59,000 rabies-associated annual deaths [4]. Therefore, developing effective and safe live vaccines that reduce rabies mortality and facilitate routine vaccination remains a priority [5], especially in light of the WHO “Zero by 2030” goal. Consequently, understanding viral determinants of pathogenicity and antiviral immunity is crucial for improving vaccination strategies and combating rabies.

**Figure 1:**
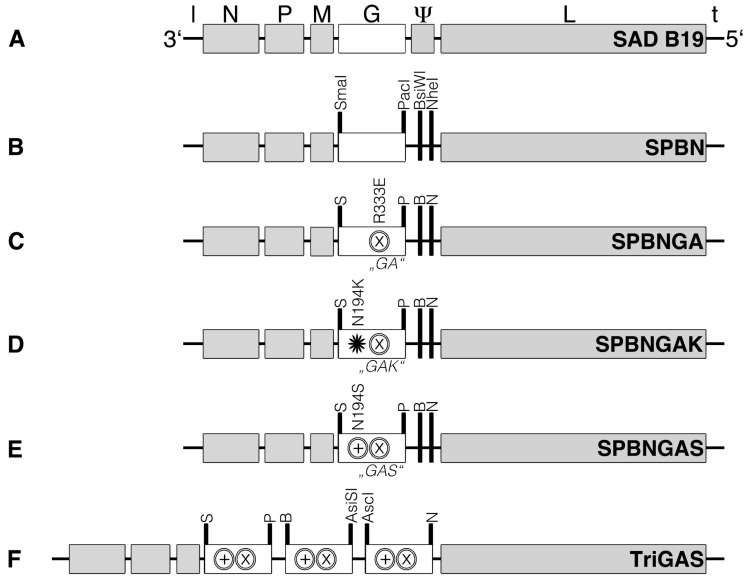
Schematic representation of the recombinant RABV constructs used in this study. (**A**) SAD B19, the parent RABV strain. (**B**) SPBN, a recombinant derivative of SAD B19 with the pseudogene (Ψ) deleted, the glycoprotein (G) flanked by two restriction sites (SmaI, S; PacI, P), and two additional restriction sites (BsiWI, B; NheI, N) in the intergenic region between G and the RNA-dependent RNA polymerase gene (L). (**C**) SPBNGA, an apathogenic but unstable variant of SPBN, harboring a single mutation (R333E, ⊗) in the glycoprotein G (“GA”). (**D**) SPBNGAK, a pathogenic variant of SPBN with two mutations in G: N194K (✹) and R333E (⊗) (“GAK”). (**E**) SPBNGAS, an apathogenic and stable variant of SPBN with two mutations in G: N194S (⊕) and R333E (⊗) (“GAS”). (**F**) TriGAS, an apathogenic and highly stable SPBN variant carrying three copies of the G “GAS” gene inserted between the restriction sites S and P, B and AsiSI, and AscI and N.

RABV is a negative-sense, single-stranded RNA virus encoding five proteins (Figure 1A). The nucleoprotein (N) encapsulates the viral genome [6], while the phosphoprotein (P) serves as a cofactor for the viral polymerase [7] and inhibits type I interferon signaling [8]. The matrix protein (M) bridges the ribonucleoprotein (RNP) core to the viral envelope and plays a central role in budding [9]. The glycoprotein (G), anchored as trimeric spikes in the viral membrane [10], mediates host cell recognition [11] and immune activation [12]. Finally, the RNA-dependent RNA polymerase (L) completes the viral machinery. Wild-type strains contain an additional pseudogene between the G and L genes, though its function remains unclear [13,14].

Wild-type RABV strains are characterized by neuroinvasiveness, retrograde trans-synaptic spread, and neurotropism. RABV G is a central contributor to these interconnected processes and thereby critically determines disease outcome [15]. Thus, elucidating how changes in the RABV G protein modulate viral tropism and immune evasion is pivotal for the design of next-generation vaccines.

Cellular tropism depends on receptor expression and cellular permissiveness [16]. Neurons, expressing multiple RABV G receptors, are prime targets in vivo: nicotinic acetylcholine receptor (nAChR) [17], p75 neurotrophin receptor (p75NTR) [18], neural cellular adhesion molecule (NCAM) [19], and metabotropic glutamate receptor (mGluR) 2 [20]. This enables invasion from peripheral sites to the CNS via axonal transport, replication in an immunoprivileged environment, and retrograde spread to the salivary glands for transmission by biting. However, no single receptor fully accounts for neuroinvasion, and the functional interplay among RABV receptors remains unclear. Additionally, non-neuronal cells, including astrocytes, can be infected in vivo, resulting in either abortive or productive infection [21,22,23].

Multiple amino acid residues in RABV G, such as positions 83 [24], 255 [25], 330 [26], 333 [27], and 349 [28], have been implicated in dictating cell tropism, although none is solely responsible. For instance, Itakura et al. [27] demonstrated that substituting arginine at position 333 with glutamic acid confers astrocyte tropism in vitro and in vivo, whereas the wild-type arginine enforces stricter neurotropism.

Many of these tropism-altering mutations also affect pathogenicity in vivo [29]. Replacing the arginine or lysine at position 333 in virulent RABV strains with, for example, glycine [30], glutamine [31,32,33,34], or leucine [35] attenuates the virus. Mutations within amino acids 194–197 of RABV G, which interact with the nAChR alpha subunit [36], altered pathogenicity without affecting tropism [37]. Mechanistically, pathogenicity-associated mutations in RABV G often enhance apoptosis by upregulating RABV G expression [12,24], or they alter neurovirulence by disrupting receptor binding [38] or reducing cell-to-cell spread [39]. In addition, variations in RABV G can alter the antiviral immune response, as the glycoprotein is the primary target of virus-neutralizing antibodies (VNA). When coupled with increased blood-brain barrier (BBB) permeability [40,41,42], VNA can eliminate RABV from the CNS. Consequently, vaccine development efforts have focused on optimizing the VNA generation while improving the safety profile of vaccine candidates by engineering RABV G and its expression [43].

Live vaccines are particularly promising because of their cost-effectiveness and simpler logistics [44], which would especially benefit regions endemic to rabies. Attenuated RABV strains bearing multiple G genes (“double G” or “triple G” constructs) generally remain nonpathogenic in adult mice [12,45] and induce neutralizing responses that protect animals from lethal challenges [46,47]. Incorporating a third copy of the G gene “GAS”—harboring the R333E pathogenicity determinant mutation and a N194S substitution in the nAChR binding motif [37]—in the SPBN-based [48] RABV platform further increased vaccine safety: “TriGAS” [49] is apathogenic even in young or immunocompromised animals [50].

Our study investigated how specific glycoprotein mutations affect RABV infection patterns and immune responses in mouse brains and disease outcomes. Prior studies have shown that substituting arginine at position 333 with glutamic acid attenuates RABV and enables astrocyte infection, while modifications at position 194 affect pathogenicity. However, the combined effect of these substitutions on astrocytotropism or CNS immunity remains unclear. Therefore, we examined recombinant RABV variants carrying distinct glycoprotein substitutions at these key positions, R333E and N194K/S, in an intracerebral (i.c.) mouse infection model. Specifically, we examined whether these mutations jointly yield astrocyte-restricted infection, glial activation, and T-cell infiltration. We also assessed whether astrocyte activation markers, such as nestin, are upregulated and whether an astrocytotropic variant affects a pathogenic RABV strain in co-infections. We hypothesized that promoting astrocyte tropism in RABV would accelerate the early immune response and subsequent viral clearance, thus mitigating its pathogenicity. We present evidence that combined R333E and N194S substitutions in RABV G markedly reduce neuronal infection and are associated with abortive astrocyte infection and survival after a concurrent virulent challenge. These findings highlight the potential of safer live RABV vaccines or experimental strategies aimed at reducing rabies lethality.

## 2. Materials and Methods

### 2.1. Viruses and Cell Lines

**Figure A1:**
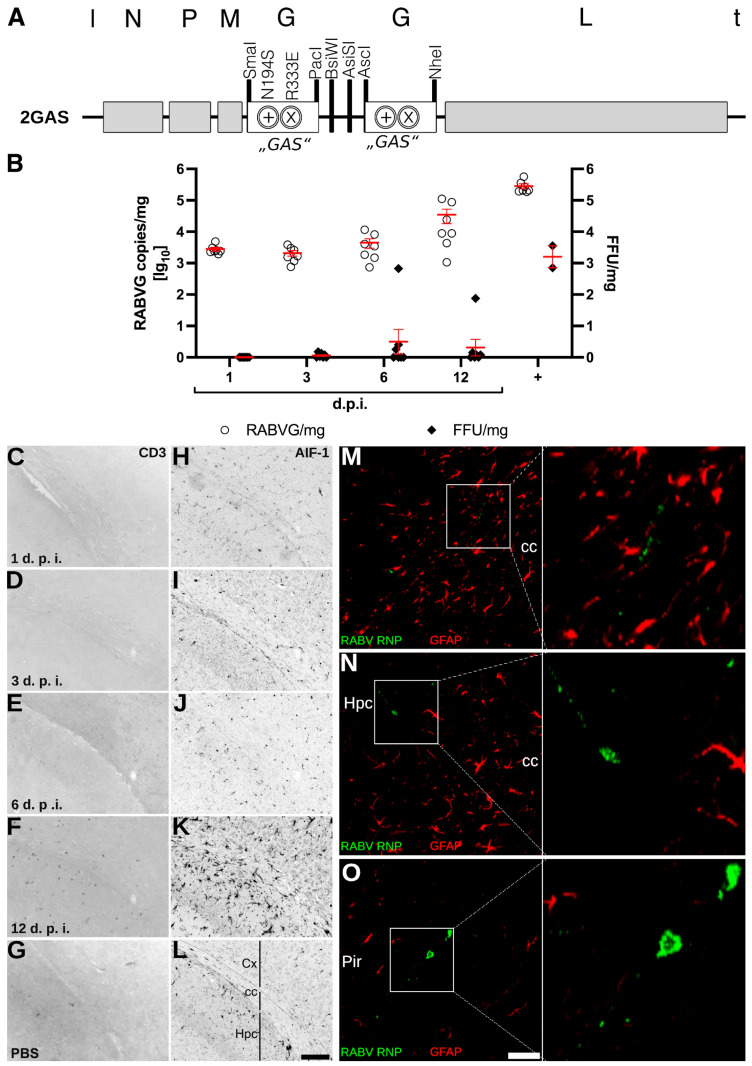
Brains of mice after intranasal inoculation with 2GAS. (**A**) Schematic representation of the recombinant RABV construct 2GAS, an apathogenic and stable SPBN variant carrying two copies of the glycoprotein gene “GAS” inserted between the restriction sites SmaI and PacI and AscI and NheI, respectively. (**B**) Viral load in mouse brains at 1, 3, 6, and 12 days post-inoculation (d.p.i.) via the intranasal (i.n.) route with 10^6^ focus-forming units (FFUs) of 2GAS. Brains were sagittally halved; one half was used for RNA isolation to quantify RABV genome (RABVG) copies per mg tissue by quantitative polymerase chain reaction (qPCR) (left y-axis; open circles; n = 7 per time point and positive control) and the other half was used for quantifying infectious particles (right y-axis; black diamonds; n = 7 per time point, n = 2 for the positive control). As a positive control (+), brain halves from uninfected mice were homogenized in the presence of 3.33 × 10^3^ FFUs 2GAS per mg tissue and processed identically to the brain samples from inoculated mice. The red lines represent the mean ± standard error of the mean (SEM). (**C**–**L**) Enzymatic immunohistochemistry of brain sections from mice at 1 (**C**,**H**), 3 (**D**,**I**), 6 (**E**,**J**), and 12 (**F**,**K**) d.p.i. (i.n.) with 2GAS or phosphate-buffered saline (PBS) (**G**,**L**). Sections were stained for the T cell marker CD3 (**C**–**G**) and the microglia/macrophage marker allograft inflammatory factor 1 (AIF-1; **H**–**L**). Images were taken at interaural 1.10 mm/bregma −2,70 mm, showing the neocortex (Cx; upper right), hippocampus (Hpc; lower left), and corpus callosum (cc; diagonal), as indicated in (L) (scale bar: 100 µm). (**M**–**O**) Confocal images of double immunofluorescence staining of brain sections from 2GAS i.n.-inoculated mice at 12 d.p.i. RABV ribonucleoprotein (RNP)-positive cells (green) were rare. Screening focused on areas previously identified as RABV-positive after TriGAS intracerebral infection, such as the corpus callosum (cc; **M**,**N**), and regions accessible via retrograde neuronal transport from the olfactory epithelium, such as the piriform cortex (Pir; **O**). None of the RABV RNP-positive cells co-localized with the astrocyte marker glial fibrillary acidic protein (GFAP; red) (scale bar: 50 µm).

The generation of the recombinant RABV vectors (Figure 1) SPBN from SAD B19 [51], as well as the subsequent generation of the variants SPBNGA [12,31], SPBNGAK [37,52], SPBNGAS [37], and TriGAS [50], has been described elsewhere. The recombinant RABV variant 2GAS was generated from cSPBNGAS [37] by amplifying the intergenic region between the PacI and NheI restriction sites and introducing AscI and AsiSI sites between the BsiWI and NheI sites. The modified fragment was ligated into cSPBNGAS, resulting in cSPBAANGAS-GAS (Figure A1A). Infectious 2GAS particles were recovered as described previously [48]. Briefly, BSR-T7 cells [53] were transfected with cSPBAANGAS-GAS using Fugene 6 (Roche, Basel, Switzerland) and expression plasmids encoding RABV N, P, L, and G (pT7T-N, -P, -L, -G; [54]). After incubation at 37 °C with 5% CO_2_ for 3 and 8 days, the supernatants were transferred onto BSR cells [55] and monitored for infectious virus. The pathogenic wild-type RABV strain DOG4 was initially isolated as DRV4 from a human brain [56]. All recombinant RABV strains were propagated in BSR cells. DOG4 was passaged in neuroblastoma (NA) cells of A/J mouse origin [56]. Briefly, cells grown in DMEM (BSR) or RPMI 1640 (NA) supplemented with 10% fetal bovine serum were infected at a multiplicity of infection (m.o.i.) of 0.1. After one hour at 37 °C, the inoculum was removed and replaced by OptiPro SFM (Invitrogen, Karlsruhe, Germany) supplemented with 4 mM glutamine (BSR cells), or by RPMI 1640 supplemented with 0.2% bovine serum albumin (NA cells). The virus was harvested after 72 h at 34 °C. Virus yields were determined in triplicate on NA cells, as described elsewhere [57].

### 2.2. Primary Astrocyte Culture

**Figure A2:**
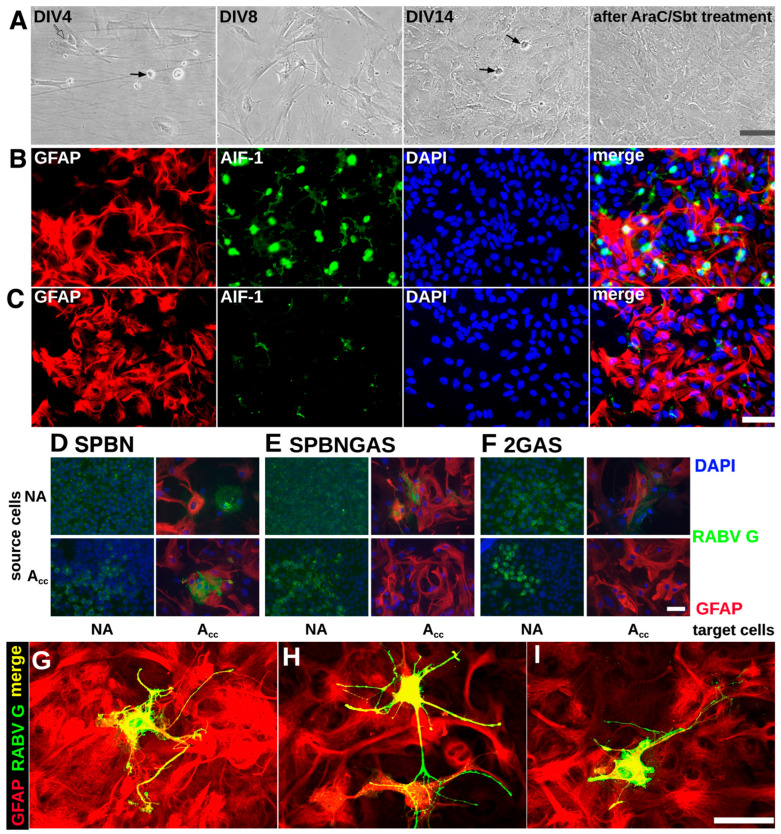
Infection of murine primary astrocytes with SPBN, SPBNGAS, and 2GAS. (**A**) Primary astrocyte cultures were established from mixed glial cultures of the neonatal corpus callosum (A_cc_; **A**,**D**–**F**) or cortex tissue (**B**,**C**,**G**–**I**). The tissue was digested with trypsin/DNase I, and the resulting single-cell suspensions were seeded onto T25 flasks or plastic coverslips. Solid arrows indicate fibrillary astrocytes, and the open arrow indicates a protoplasmic astrocyte. Over 14 days in vitro (DIV), protoplasmic astrocytes increased in number and surface area, whereas fibrillary astrocytes remained scarce. To minimize microglial contamination, the medium was changed every two to three days to reduce astrocyte-derived colony-stimulating factor 1 levels. After DIV14, once the cells reached confluency, three additional medium changes were performed with sorbitol (Sbt) instead of glucose to provide an energy source that could only be metabolized by astrocytes. Additionally, cytosine arabinoside (AraC) was added to induce apoptosis in dividing microglia. As astrocyte proliferation is contact-inhibited, this treatment selectively reduced the number of microglia. Scale bar, 50 µm. (**B**,**C**) Immunofluorescence staining of confluent primary astrocyte cultures without (**B**) and with (**C**) AraC/Sbt treatment using DAPI (blue), GFAP (red), and AIF-1 (green) antibodies. The scale bar in (**C**) applies to (**B**,**C**): 50 µm. To further deplete microglia from cultures intended for viral infection, flasks were shaken after each AraC/Sbt treatment to dislodge loosely attached cells. (**D**–**F**) Virus transmission assay. Supernatants from neuroblastoma (NA) cells or A_cc_ astrocytes inoculated in T25 flasks with SPBN (**D**), SPBNGAS (**E**), or 2GAS (**F**) at a multiplicity of infection (m.o.i.) of 0.01 were harvested 96 h post-infection (h.p.i.). These supernatants were used to inoculate target cells (NA and A_cc_) grown on coverslips in 24-well plates (maximum m.o.i. 5). After two days, the cells were stained with DAPI (blue), GFAP (red), and RABV G (green) and analyzed using epifluorescence microscopy. The scale bar in (**F**) applies to (**D**–**F**): 50 µm. (**G**–**I**) Confocal images of fibrillary astrocytes in primary astrocyte cultures two days after infection with SPBN (**G**), SPBNGAS (**H**), or 2GAS (**I**) at m.o.i. 5, stained with antibodies against GFAP (red) and RABV G (green). Notably, neighboring astrocytes in close proximity to the infected cells remained uninfected. The size bar in (**I**) applies to (**G**–**I**): 20 µm.

Primary astrocytes were prepared from the cortices or corpus callosum of 1–6-day-old TgN(hGFAP-EGFP)GFEA mice (GFAP/EGFP transgenic mice with FVB background [58]; breeding and euthanasia approved by Regierungspräsidium Gießen, Germany: V 54–19 c 20 15 (1) MR 20/26 Nr. A 15/2012) using a protocol based on the procedures described by others [59,60,61]. Tissues were mechanically dissociated using a 70 µm sieve, followed by enzymatic dissociation with 0.25% trypsin (Seromed/Biochrom, Berlin, Germany) at 37 °C for 20 min. After inactivation with DMEM-10 (DMEM 4.5 g/L glucose with 10% fetal bovine serum gold (PAA Laboratories, Cölbe, Germany), 4 mM glutamine, and penicillin/streptomycin), DNase I (Sigma-Aldrich, Seelze, Germany) was added for 10 min at 37 °C. The single-cell suspension was pelleted, washed twice, and counted using a Neubauer chamber. 1 × 10^6^ cells were seeded in Cell^+^ T25 flasks (Sarstedt, Nümbrecht, Germany) or 1–3 × 10^5^ cells on Cell^+^ 12 mm coverslips in 24-well plates (Sarstedt). Cells were maintained in DMEM-10 at 37 °C/5% CO_2_ until confluence (~14 days) (Figure A2A).

To reduce the number of microglia, cultures underwent three 2-day treatments with glucose-free DMEM supplemented with 10% FBS Gold, 4 mM glutamine, penicillin/streptomycin, 25 mM sorbitol [62,63], and 8 µM cytosine arabinoside (Sigma-Aldrich) [64]. Efficiency was confirmed by reverse transcription-polymerase chain reaction (RT-PCR) for the microglial marker gene colony-stimulating factor 1 receptor and immunocytochemistry for AIF-1 (Figure A2B,C). Astrocytes on coverslips were then used for immunocytochemical analyses; astrocytes in flasks were shaken for 30 min at 180 rpm on a rotary shaker at 4 °C to further reduce microglial contamination before use in viral growth curve experiments.

### 2.3. One-Step and Multi-Step Growth Curves

**Figure A3:**
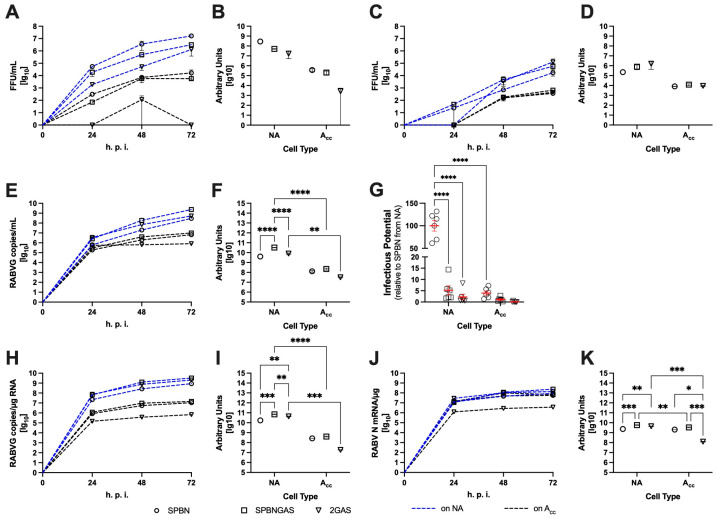
Comparative growth analysis of SPBN, SPBNGAS, and 2GAS in NA cells and primary astrocytes from the murine corpus callosum. (**A**) One-step growth curves (m.o.i. 10) of SPBN (circles), SPBNGAS (squares), and 2GAS (triangles) in NA cells (blue lines) and primary astrocytes from the murine corpus callosum (A_cc_, black lines). Viral titers were determined in duplicate on BSR cells at 24, 48, and 72 h.p.i. (**B**) Area under the curve (AUC) analysis of the data shown in (**A**). Data points represent the mean ± SEM. Two-way ANOVA: *p* = 0.0260 (cell type), *p* = 0.0651 (virus strain), and *p* = 0.0656 (interaction). (**C**) Multi-step growth curves (m.o.i. 0.01) of SPBN (circles), SPBNGAS (squares), and 2GAS (triangles) in NA cells (blue lines) and A_cc_ (black lines). Viral titers were determined in duplicate on BSR cells at 24, 48, and 72 h.p.i. (**D**) AUC analysis of the data shown in (**C**). Two-way ANOVA: *p* > 0.05 (cell type, virus strain, and interaction). (**E**) qPCR analysis of RABVG RNA in the supernatants of NA cells (blue lines) and A_cc_ (black lines) infected at an m.o.i. of 10 with SPBN (circles), SPBNGAS (squares), or 2GAS (triangles). Samples were collected at 24, 48, and 72 h.p.i. and RABVG copies were quantified in triplicate using cDNA generated with RABVG-specific primers. Data points represent the mean ± SEM from the same two experiments as in (**A**). (**F**) AUC analysis of the data shown in (**E**). Two-way ANOVA: *p* < 0.0001 (cell type, viral strain, interaction). Šídák’s multiple-comparison test results are displayed. (**G**) Infectious potential of virus particles in the supernatants from NA cells and A_cc_ after infection with SPBN (circles), SPBNGAS (squares), or 2GAS (triangles) at m.o.i. 10. The infectious potential was calculated as the ratio of FFU to RABVG copies in six samples for each virus and cell type. The values were normalized to the mean infectious potential of SPBN-infected NA cells. Red lines indicate the mean ± SEM. Two-way ANOVA: *p* < 0.0001 (cell type, virus strain, interaction). The results of Šídák’s multiple comparisons test are shown. (**H**) qPCR analysis of RABVG in RNA extracted from NA cells (blue lines) and A_cc_ (black lines) infected at an m.o.i. of 10 with SPBN (circles), SPBNGAS (squares), or 2GAS (triangles) and sampled at 24, 48, and 72 h.p.i. RABVG copies were quantified in triplicate using cDNA generated with RABVG-specific primers. (**I**) AUC analysis of (**H**). Two-way ANOVA: *p* < 0.0001 (cell type), *p* = 0.0004 (virus strain, interaction). The results of Šídák’s multiple comparisons test are shown. (**J**) qPCR analyses of RABV nucleoprotein (N) mRNA in cellular RNA from NA cells (blue lines) and A_cc_ (black lines) infected at an m.o.i. of 10 with SPBN (circles), SPBNGAS (squares), or 2GAS (triangles) and sampled at 24, 48, and 72 h.p.i. RABV N mRNA copies were quantified in triplicate from cDNA generated using oligo-dT primers. Data points represent the mean ± SEM from the same two experiments as in (**H**). (**K**) AUC analysis of (**J**). Two-way ANOVA: *p* < 0.0001 (cell type, virus strain), *p* = 0.0005 (interaction). The results of Šídák’s multiple comparisons test are shown. Data points in (**A**–**F**) and (**H**–**K**) represent mean ± SEM from two independent experiments. qPCR reactions were performed in technical triplicate, virus titrations in technical duplicate per condition. RABVG and RABV N mRNA copy numbers were quantified using standard curves with known concentrations. Statistical significance: *, *p* ≤ 0.05; **, *p* ≤ 0.01; ***, *p* ≤ 0.001; ****, *p* ≤ 0.0001.

Growth curves were generated on primary astrocytes from the corpus callosum and NA cells for comparison. To standardize the cell numbers, parallel cultures were trypsinized and counted at the time of inoculation. For one-step growth curves (m.o.i. 10; Figure A3A,B) and multi-step growth curves (m.o.i. 0.01; Figure A3C,D), cells were infected in OptiPro SFM (astrocytes) or Advanced RPMI (NA cells) (both media: Invitrogen; supplemented with 4 mM glutamine and penicillin/streptomycin) for 1 h at 37 °C at 5% CO_2_ before washing and replacing media with DMEM-10 (astrocytes) or RPMI-10 (NA cells).

Supernatants were collected for virus titration (100 µL) and RNA isolation (140 µL) at 24, 48, and 72 h (one-step) and 24, 48, 72, and 96 h (multi-step). The remaining multi-step supernatants at 96 h were stored for later analysis. After supernatant collection, the one-step cultures were trypsinized, washed, and lysed for RNA isolation.

### 2.4. Infection of Cells for Immunocytochemistry

Supernatants from multi-step growth curves were collected at 96 h.p.i., titrated, and used to infect NA cells and primary astrocytes on 12 mm coverslips at a maximum m.o.i. of 5. After 1 h at 37 °C/5% CO_2_, the cells were washed, fed with RPMI-10 (NA) or DMEM-10 (astrocytes), and incubated for 48 h at 37 °C/5% CO_2_. Cells were then fixed with 80% acetone at −70 °C for 10 min, stored at 4 °C, and stained with DAPI and for glial fibrillary acidic protein (GFAP) and RABV glycoprotein (G) antibodies (Table A1; Figure A2D–I). Cell staining was documented using an AX70 microscope (Olympus, Hamburg, Germany) or an Eclipse Ti2 inverted microscope with a C2 confocal system (Nikon, Düsseldorf, Germany).

### 2.5. Intracerebral Infection of Mice

Fifty-seven female Swiss Webster mice (6–8 weeks old) were purchased from the National Cancer Institute (Frederick, MD, USA). All mice in a single cage were treated uniformly, and the cages were randomly assigned for treatment. Mice were kept under a 12L:12D light-dark cycle, with food and water provided ad libitum. Throughout the study, n refers to the number of animals used. The sample size was chosen so that the resource equation E [65] was greater than 10. Mice were injected i.c. under isoflurane anesthesia with 5 µL phosphate-buffered saline (PBS) containing nothing else (control group; n = 3); 10^3^ focus forming units (FFUs) of SPBN (n = 3), SPBNGA (n = 6), SPBNGAK (n = 3), SPBNGAS (n = 6), or TriGAS (n = 16); 100 50% i.c. lethal doses (IC-LD50) of DOG4 RABV (n = 10); or a mixture of 10^7^ FFU TriGAS and 100 IC-LD50 of DOG4 RABV (n = 10). Treated mice were monitored daily for symptoms, and all were euthanized before the appearance of disease-specific symptoms. At different time points after intracerebral infection (comparison of SPBN variants: 3 days post-infection (d.p.i.) and 20 d.p.i., E = 18 with n = 3 per experimental group and time point; time course experiment: 1–5 d.p.i., E = 17 with n = 2 per treatment and time point), tissues were harvested and processed as previously described [37]: Mice were anesthetized and perfused transcardially with PBS containing procaine-HCl (5 g/L) and heparin (20,000 IU/L), followed by Bouin-Hollande fixation solution. The brains were removed and post-fixed for 24 h in the same fixative. The control group was used in both experiments. All animals were included in the analysis. Experiments were performed at Thomas Jefferson University under Institutional Animal Care and Use Committee-approved protocols (Animal Welfare Assurance no. A3085–01).

### 2.6. Intranasal Infection of Mice

Female Swiss Webster mice (7 weeks old) (Charles River, Sulzfeld, Germany) were acclimated for 1 week before intranasal (i.n.) inoculation with 10^6^ FFUs 2GAS in 10 µL PBS under isoflurane anesthesia. Mice were scored daily (0 = healthy to 5 = moribund) based on piloerection, trunk curl test, righting and reaching reflexes, and body condition score [66]. No symptoms were observed, and all animals had a score of 0 until euthanasia on days 1, 3, 6, and 12 post-inoculation.

For tissue analysis, the brains of seven mice per timepoint and control group were sagittally halved:

- Left hemispheres: storage at −70 °C until virus isolation (Figure A1B).
- Right hemispheres: immersion in RNAlater (Qiagen, Hilden, Germany) at room temperature overnight, then storage at −70 °C until RNA isolation for subsequent RT and quantitative PCR (qPCR) (Figure A1B).

The brains of three additional mice per timepoint and control group were fixed in Bouin Hollande solution for 48 h, paraffin-embedded, and sectioned for immunohistochemistry (Figure A1C–L) and immunofluorescence (Figure A1C,M–O).

The procedures were approved by the Regierungspräsidium Gießen (V 54—19 c 20 15 h 01 MR 20/26 122/2012).

### 2.7. Immunohistochemical and Immunofluorescence Analyses

All tissues fixed in Bouin-Hollande solution were dehydrated in a graded series of 2-propanol and embedded in Paraplast Plus (Merck, Darmstadt, Germany). Immunohistochemical staining was performed on 7 µm thick coronal sections through the hippocampus (Bregma −1.06 mm–−1.58 mm), as previously described [67]. For enzymatic immunostainings, sections were incubated with (i) a polyclonal rabbit antibody raised against the RABV RNP complex, diluted 1:3000 (source: B. Dietzschold) [68]; (ii) a polyclonal rabbit antibody raised against RABV G, diluted 1:3000 (source: B. Dietzschold) [69]; (iii) a polyclonal rabbit-anti-CD3 (catalog no. A0452; Dako Deutschland GmbH, Hamburg, Germany) to localize T lymphocytes, diluted 1:2500; (iv) a polyclonal rabbit anti-ionized Ca2+-binding adapter (IBA) 1 (catalog no. 019-19741; Wako Pure Chemical Industries, Ltd., Osaka, Japan) to detect allograft inflammatory factor 1 (AIF-1) in macrophages and microglial cells, diluted 1:2000; (v) a polyclonal guinea pig anti-glial fibrillary acidic protein (GFAP) (clone GP52, Progen Biotechnik GmbH, Heidelberg, Germany) to label astrocytes, diluted 1:3000; and (vi) a polyclonal rabbit anti-activated caspase 3 antibody to detect apoptotic cells, diluted 1:500 (BD Pharmingen, San Diego, CA, USA). Staining was analyzed using an Olympus AX70 microscope (Olympus Optical, Hamburg, Germany) or the Axio Imager M2 system (Carl Zeiss, Oberkochen, Germany). For all immunohistochemical and immunofluorescence stainings, uninfected brain tissue processed in parallel served as negative controls and consistently showed no specific signal (RABV RNP, G) or the expected baseline staining patterns (AIF-1, CD3, SELP, nestin). For double immunofluorescence analyses, uninfected tissue and single-label controls were evaluated during assay optimization and showed no unspecific signal overlap or staining patterns different from those observed in enzymatic immunohistochemistry of the same tissue samples using the same primary antibodies.

For confocal immunofluorescence microscopy, the following primary antibodies were applied singly or mixed in pairs: (i) the polyclonal rabbit-anti-RABV RNP, diluted 1:300; (ii) the polyclonal rabbit antibody raised against RABV G, diluted 1:250 on tissue and 1:500 on cells; (iii) the polyclonal guinea pig-anti-GFAP (Progen), diluted 1:300; (iv) a polyclonal rabbit-anti-P-selectin (SELP) for labeling activated endothelial cells, diluted 1:50 (BD Pharmingen); (v) a monoclonal mouse-anti-nestin antibody, diluted 1:200 (MAB353, clone Rat-401, Millipore GmbH, Schwalbach/Ts., Germany); and (vi) a polyclonal goat-anti-C1q, diluted 1:500, was used to stain for complement deposits (a gift from Michael Loos to Eberhard Weihe, [70]). Immunofluorescence staining of brain tissue was documented as digitized false-color images obtained using an Olympus BX50WI laser-scanning microscope (Olympus Optical) or an Eclipse Ti2 inverted microscope and C2 confocal system (Nikon, Düsseldorf, Germany). Immunofluorescence staining of cells was documented using an Axiovert 200M confocal system (Carl Zeiss Microscopy, Jena, Germany). Channel colors shown in the figures represent Alexa Fluor 647-conjugated secondary antibodies (red) and Alexa Fluor 488-conjugated secondary antibodies (green), respectively, with co-localization of signals from both channels depicted in yellow. Single-channel images are shown alongside merged images for the core figures to ensure interpretability independent of color perception.

### 2.8. Virus Isolation from the Brains of Infected Mice

To determine the infectious virus loads in the brains of i.n. inoculated mice, 2 × 10^5^ BSR cells were pelleted and resuspended in 400 µL of 10% (*w*/*v*) brain homogenate prepared in OptiPro SFM supplemented with 50 µg/mL DEAE-dextran (Sigma-Aldrich). As a positive control, BSR cells were incubated with brain homogenates from uninfected mice supplemented with 3.33 × 10^3^ FFU 2GAS per mg tissue.

After 30 min of incubation at 37 °C, the cell-homogenate mixtures were pelleted, resuspended in 2 mL DMEM-10, and seeded into two wells of a 24-well plate (1 mL per well). Cells were incubated for 48 h at 37 °C/5% CO_2_, then fixed with 80% acetone at −70 °C for 10 min and stained with FITC-conjugated anti-RABV N antibody (Fujirebio Diagnostics, Seguin, TX, USA) in FAD solution (0.5% FITC-anti-RABV N, 0.00125% Evans Blue, 1% bovine serum albumin, 0.1% sodium azide in PBS) for 2 h at 37 °C.

Fluorescent foci were counted using an AX70 fluorescence microscope (Olympus), and infectious virus titers were calculated as FFUs per milligram of the original brain tissue (Figure A1B).

### 2.9. RNA Isolation and Quantitative PCR

RNA from brains and cells was isolated using the RNeasy Mini Kit (Qiagen) with DNase I treatment. RNA was extracted from the supernatants using the Viral RNA Mini Kit (Qiagen). RNA concentrations from tissues and cells were determined using Nanodrop 2000c (PEQLAB Biotechnologie, Erlangen, Germany).

For qPCR, cDNA was synthesized using the TaqMan Reverse Transcription Kit (Applied Biosystems, Waltham, MA, USA) primed either with oligo-dT for subsequent RABV nucleoprotein (N) mRNA quantification or with MP079 (Table A1) for the quantification of RABV genomes (RABVG). Copy numbers were determined using SYBR Green PCR Master Mix (Applied Biosystems) with the primer pairs MP079/MP080 (RABVG) and MP081/MP082 (RABV N mRNA) (Table A1). Standard curves for absolute quantification were generated from known RABVG and N mRNA copy numbers, derived from purified PCR amplicons and diluted in 100 ng/µL *E. coli* t-RNA over a range of 10^0^ to 10^7^ (Table A2).

### 2.10. Statistical Analysis

Data in this study are presented qualitatively or semi-quantitatively and were not subjected to numerical quantification, except for the in vitro infection shown in Figure A3. Data were analyzed using two-way ANOVA to assess the effects of cell type and virus strain, including interaction terms. All tests were two-tailed. Where indicated, Šídák’s multiple-comparison test was applied to correct for multiple testing. Assumptions of normality and variance homogeneity were not formally tested due to limited sample size; statistical analyses were therefore interpreted descriptively and in conjunction with biological reproducibility. All statistical analyses were performed using Prism 10 version 10.3 (GraphPad Software, Boston, MA, USA).

### 2.11. Software

Microsoft Word version 16.103.1 and Excel version 16.103.2 (Microsoft Corporation, Redmond, WA, USA), Prism 10 version 10.3 (GraphPad Software, Boston, MA, USA), Zotero version 7.0.30 (Corporation for Digital Scholarship, Vienna, VA, USA), ImageJ2 version 2.16.0 [71], Grammarly Pro version 1.136.4.0 (Superhuman Platform, San Francisco, CA, USA), Paperpal Prime (Cactus Communications Services, Singapore), ChatGPT version GPT-5 (OpenAI Ireland, Dublin, Ireland), and Photopea version 5.6 (Kutskir, Ivan (2025) www.photopea.com).

## 3. Results

### 3.1. Amino Acid Substitutions at Position 194 of the Rabies Virus Glycoprotein Are Associated with Altered Virus Distribution in Mouse Brains After Intracerebral Injection

**Figure 2:**
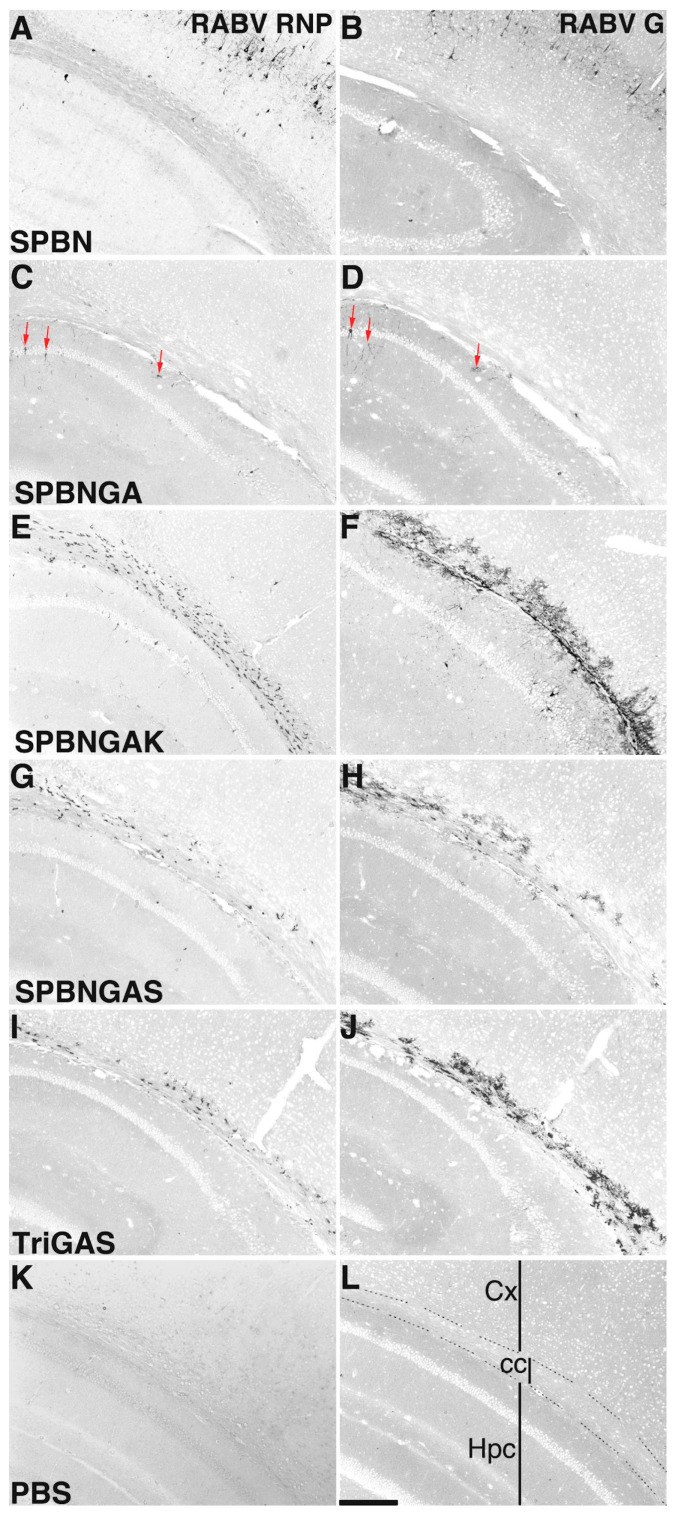
RABV G and RABV RNP localization in brain sections following intracerebral (i.c.) infection with different SPBN variants. Enzymatic immunohistochemical staining was performed on one to four sections from two to four animals per experimental group to detect RABV ribonucleoprotein (RNP; left column). Immunostaining of RABV glycoprotein (G; right column) is displayed on consecutive sections. Brains were collected 3 d.p.i. (i.c.) with SPBN (**A**,**B**), SPBNGA (**C**,**D**), SPBNGAK (**E**,**F**), SPBNGAS (**G**,**H**), TriGAS (**I**,**J**), or phosphate-buffered saline (PBS; (**K**,**L**)). No viral antigen was detected in the control group with either of the two anti-RABV antibodies (**K**,**L**). As outlined in (**L**), all sections display the hippocampus (Hpc) in the lower left corner, neocortex (Cx) in the upper right corner, and the corpus callosum (cc) diagonally in between. The red arrows in (**C**,**D**) indicate neurons that showed positive staining for both RNP and G. The scale bar in (**L**) represents 200 µm.

In the forebrain at 3 d.p.i. (i.c.), the parental strain SPBN primarily infected neocortical neurons, particularly in layers III and V (Figure 2A,B). The apathogenic SPBNGA variant differs from SPBN by an R333E substitution in its glycoprotein. It exhibited a reduced viral load, with only sparse neurons in the hippocampus showing viral protein expression (Figure 2C,D).

The additional N194K substitution in SPBNGAK was associated with higher viral loads compared with the R333E mutation alone. SPBNGAK exhibited a distinct distribution pattern in the forebrain: It infected neocortical cells, similar to SPBN, and hippocampal cells, like SPBNGA, but also cells in the corpus callosum (Figure 2E,F).

Substituting lysine at position 194 of the RABV G with serine (SPBNGAS) further altered the virus distribution: only cells in the corpus callosum (Figure 2G,H) and cells near the lateral and third ventricles exhibited immunoreactivity.

In TriGAS-infected brains, the viral load (Figure 2I) was comparable with that of SPBNGAS (Figure 2G), but RABV G immunoreactivity was more prominent (Figure 2J compared with H).

These observations suggested that the amino acid substitution at position 194 of the RABV G was associated with distinct viral distribution patterns in the i.c.-infected mouse brain.

### 3.2. Amino Acid Substitutions at Position 194 of the Rabies Virus Glycoprotein Are Associated with Altered Cellular Tropism Patterns

**Figure 3:**
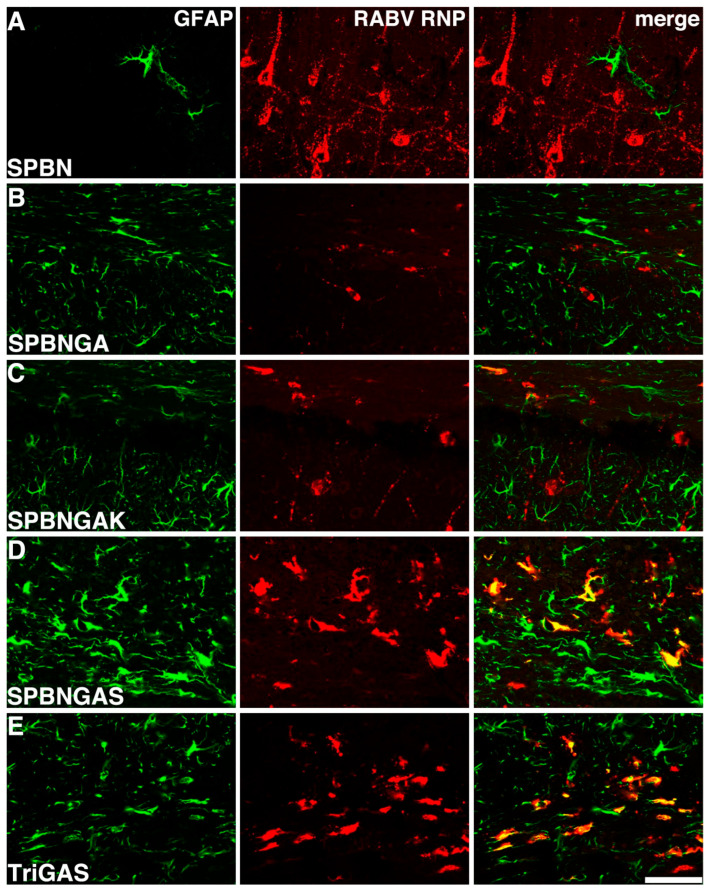
The tropism shift of RABV variants correlates with amino acid substitutions at position 194 of the glycoprotein. Immunofluorescent images display co-staining for RABV ribonucleoprotein (RNP; red) and the astrocyte marker glial fibrillary acidic protein (GFAP; green) in brain sections (representative for one to four sections from one to two animals per experimental group) from mice at 3 d.p.i. (i.c.) with SPBN (**A**), SPBNGA (**B**), SPBNGAK (**C**), SPBNGAS (**D**), or TriGAS (**E**). The images depict infected cells in the neocortex (**A**), at the border between the corpus callosum and hippocampus (**B**,**C**) and within the corpus callosum (**D**,**E**). The co-localization of RABV RNP and GFAP appears yellow in the merged images. Scale bar: 50 µm.

Neuronal somata are absent in the corpus callosum, and the morphology of virus-infected cells was consistent with that of fibrous astrocytes. To determine cell identity, we performed double immunofluorescence staining for RABV ribonucleoprotein (RNP) and the astrocyte marker glial fibrillary acidic protein (GFAP). This approach enabled qualitative assessment of infected cell identity rather than quantification of infection rates or replication efficiency (Figure 3). SPBN infection resulted in exclusive neuronal tropism without RNP-GFAP co-localization (Figure 3A). Similarly, brains infected with SPBNGA, which carries the R333E mutation in its glycoprotein, exhibited no overlap between GFAP- and RNP-immunoreactive cells. This included RNP-positive nerve fibers in the corpus callosum and RNP-positive hippocampal neurons (Figure 3B). However, in brains infected with the pathogenic SPBNGAK variant, carrying the additional N194K mutation, GFAP-negative cells were infected in the neocortex and hippocampus. In contrast, all infected cells in the corpus callosum were GFAP-positive (Figure 3C). In comparison, SPBNGAS and TriGAS, which bear the N194S mutation in RABV G, were associated with an astrocyte-restricted infection pattern in the corpus callosum (Figure 3D,E). This shift in the tropism of GAS-expressing variants depended on the route of inoculation. After intranasal (i.n.) inoculation, 2GAS (Figure A1A) caused no clinical symptoms, and brain infection was minimal (Figure A1B,M–O). No infected GFAP-positive cells were detected (Figure A1M–O). Moreover, SPBNGAS and 2GAS did not significantly differ from SPBN in replication, transcription, viral titers, or infectivity of released viral particles in primary murine astrocyte cultures (Figure A2 and Figure A3).

### 3.3. Immune Responses to RABV Infection Differ Based on the Amino Acids at Positions 333 and 194 of the Glycoprotein

**Figure 4:**
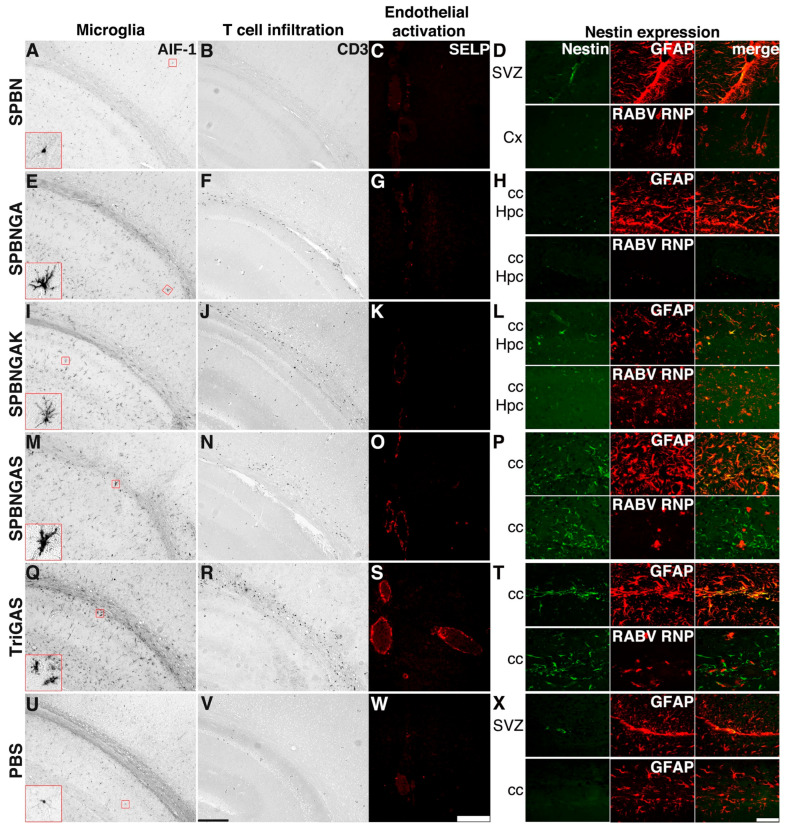
Cellular responses in the brain following i.c. infection with different SPBN variants. Representative immunostainings of brain sections (one to four sections per staining from one to five animals per experimental group) collected 3 d.p.i. with SPBN (**A**–**D**), SPBNGA (**E**–**H**), SPBNGAK (**I**–**L**), SPBNGAS (**M**–**P**), TriGAS (**Q**–**T**), or PBS (**U**–**X**). **Left column**: Enzymatic immunohistochemical staining for the microglial marker allograft inflammatory factor 1 (AIF-1). Red insets highlight the higher magnification of microglial cells within RABV-positive areas (inset size: 20 µm). **Second column**: Enzymatic immunohistochemical staining for the T-cell marker CD3. The sections of both columns represent the hippocampus (lower left), neocortex (upper right), and corpus callosum (diagonal) (scale bar: 200 µm). **Third column**: Immunofluorescent staining for P-selectin (SELP), an endothelial activation marker, showing venous vessels in the midline above the corpus callosum (scale bar: 50 µm). **Right column**: Immunofluorescent co-staining for nestin (green), a type VI intermediate filament protein expressed in neural stem cells and reactive astrocytes, and the astrocyte marker glial fibrillary acidic protein (GFAP; red) or RABV ribonucleoprotein (RNP; red). Co-localization appears yellow in the merged images (scale bar: 50 µm). Abbreviations: SVZ, subventricular zone; Cx, neocortex; cc, corpus callosum; Hpc, hippocampus.

Different cellular infection patterns among RABV variants were accompanied by qualitatively different local immune responses. Immunohistochemical analysis of allograft inflammatory factor 1 (AIF-1), a microglial/macrophage marker, revealed widespread microglial activation at 3 days post-i.c. injection. This was observed in all brains infected with variants carrying the R333E mutation (Figure 4E,I,M,Q). Conversely, microglia in SPBN-infected brains remained in a homeostatic state, as indicated by their delicate branching patterns (Figure 4A: inset), similar to those in the PBS-injected controls (Figure 4U: inset).

The R333E mutation was consistently associated with microglial activation. However, the microglial morphology varied with the glycoprotein residue at position 194. Asparagine (SPBNGA) and lysine (SPBNGAK) were associated with ramified hypertrophic microglia (Figure 4E,I: insets), whereas serine (GAS variants) coincided with the detection of amoeboid microglial cells (Figure 4M,Q: insets). This morphological transformation was consistently observed in TriGAS-infected brains (Figure 4Q, inset).

Cerebral T-cell infiltration was also affected by glycoprotein modifications. T cells were absent after SPBN i.c. infection (Figure 4B), similar to PBS controls (Figure 4V). In contrast, T-cell infiltration was observed after infection with all R333E-containing variants (Figure 4F,J,N,R). After GAS variant infection, endothelial activation was seen in larger cerebral venules and veins, as indicated by SELP immunostaining (Figure 4O,S).

**Figure 5:**
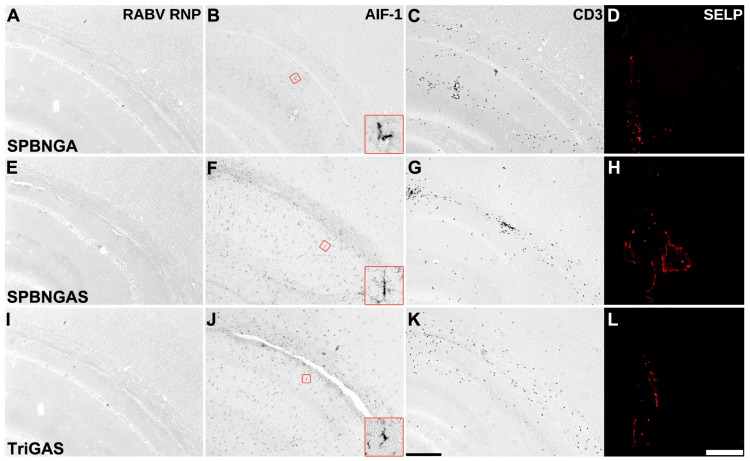
Persistence of cellular responses following virus clearance after infection with apathogenic SPBN variants. Immunohistochemical staining for RABV ribonucleoprotein (RNP; **left column**), the microglial marker allograft inflammatory factor 1 (AIF-1; **second column**), and the T cell marker CD3 (**third column**), along with immunofluorescent staining for SELP (**right column**), a marker for endothelial activation, in brain sections (one to two sections per staining from one to two animals per experimental group) collected at 20 d.p.i. (i.c.) with SPBNGA (**A**–**D**), SPBNGAS (**E**–**H**), or TriGAS (**I**–**L**) is shown. The enzymatically stained sections display the hippocampus (lower left) and neocortex (upper right), with the corpus callosum running diagonally (scale bar: 200 µm). Insets in the second column show higher magnifications of microglial cells from RABV-positive areas observed at 3 d.p.i. (inset size: 20 µm). The images in the right column highlight larger venous vessels in the midline above the corpus callosum (scale bar: 50 µm).

By 20 d.p.i., viral proteins were undetectable in the brains infected with SPBNGA, SPBNGAS, or TriGAS (Figure 5A,E,I). However, endothelial activation was observed in cerebral veins from all previously infected mice (Figure 5D,H,L), and microglial activation persisted (Figure 5B,F,J). Parenchymal T cells were also still present at this later time point in the brains of surviving mice (Figure 5C,G,K). In contrast to i.c. inoculation with GAS variants, i.n. inoculation of 2GAS was associated with delayed CNS immune activation: T-cell infiltration (Figure A1C–F) and microglial activation (Figure A1H–K) were not detected at 1, 3, and 6 d.p.i., but were apparent at 12 d.p.i. At this time point, the microglia exhibited ramified hypertrophic morphology like that seen in SPBNGA- and SPBNGAK-infected brains after i.c. inoculation.

### 3.4. Astrocyte Activation Depends on the Amino Acid at Position 194 of the Rabies Virus Glycoprotein

To evaluate astrocyte activation, we performed double immunofluorescence for nestin, a marker of reactive astrocytes and neural progenitor cells, along with RABV RNP or GFAP. PBS-injected controls showed only a few GFAP/nestin-co-stained (presumably progenitor) cells in the subventricular zone (Figure 4X: SVZ) and none in the corpus callosum (Figure 4X: cc). After SPBN inoculation, we detected no nestin expression in the neocortex surrounding infected pyramidal neurons (Figure 4D: Cx). In contrast, SPBNGAS and TriGAS inoculation resulted in nestin upregulation in GFAP-positive corpus callosum astrocytes (Figure 4P,T: upper panels). However, the infected cells did not exhibit nestin expression (Figure 4P,T: lower panels). In SPBNGA-infected brains, nestin upregulation in GFAP-positive cells was limited (Figure 4H). SPBNGAK inoculation upregulated nestin in GFAP-positive cells, including those harboring viral antigen.

In summary, astrocytes upregulated nestin expression in regions containing RABV-positive cells after infection with any of the SPBN variants carrying the R333E substitution. However, the amino acid at position 194 correlated with distinct nestin expression patterns in virus-positive astrocytes: serine (SPBNGAS, TriGAS) was associated with mutual exclusion of nestin and viral immunoreactivity. In contrast, lysine (SPBNGAK) was linked to nestin expression in virus-positive GFAP-expressing cells. Accordingly, nestin expression was detected in astrocytes infected with SPBNGAK, but not in those infected with GAS variants.

### 3.5. Astrocytotropic Rabies Virus Variant TriGAS Defines Infection and Immune Response Patterns During Mixed Intracerebral Infection

To assess phenotypic consequences associated with astrocyte-restricted infection, we compared infection patterns in mice inoculated i.c. with the highly pathogenic RABV strain DOG4 alone, TriGAS alone, or a DOG4-TriGAS mixture.

**Figure 6:**
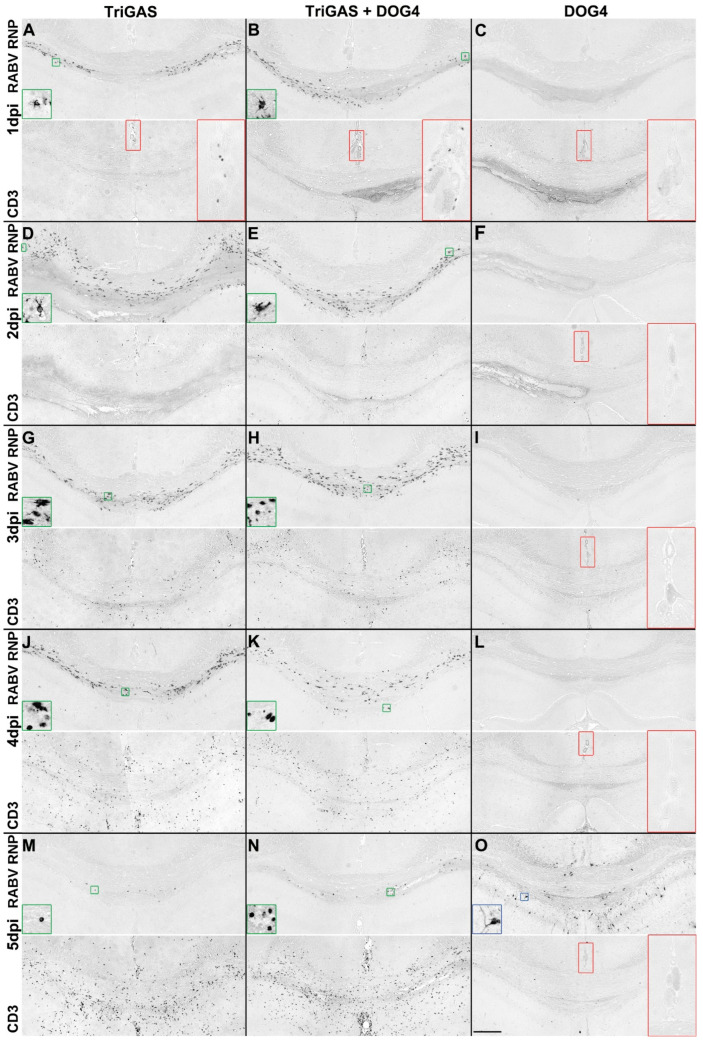
Co-infection with TriGAS was associated with T-cell infiltration and restricted DOG4 spread in the brain. Representative immunohistochemical staining of brain sections (one to thirteen sections per staining from one to two animals per time point and experimental group) for RABV ribonucleoprotein (RNP; upper half of each panel) and the T cell marker CD3 (lower half of each panel) at 1–5 d.p.i. (i.c.) (from top to bottom) with TriGAS alone (**left column:** (**A**,**D**,**G**,**J**,**M**)), DOG4 alone (**right column:** (**C**,**F**,**I**,**L**,**O**)), or a combination of TriGAS and DOG4 (**middle column:** (**B**,**E**,**H**,**K**,**N**)). All images were stitched from two fields of view of the same brain section. The sections depict the corpus callosum as an upward arch, with parts of the hippocampus below and the neocortex above (scale bar: 200 µm). Insets in the upper half of each panel show higher magnifications of RABV RNP-immunoreactive cells, with green insets indicating astrocytes and blue insets indicating neurons (inset size: 50 µm). The red insets in the lower half highlight larger venous vessels in the midline above the corpus callosum (inset width: 100 µm).

**Figure 7:**
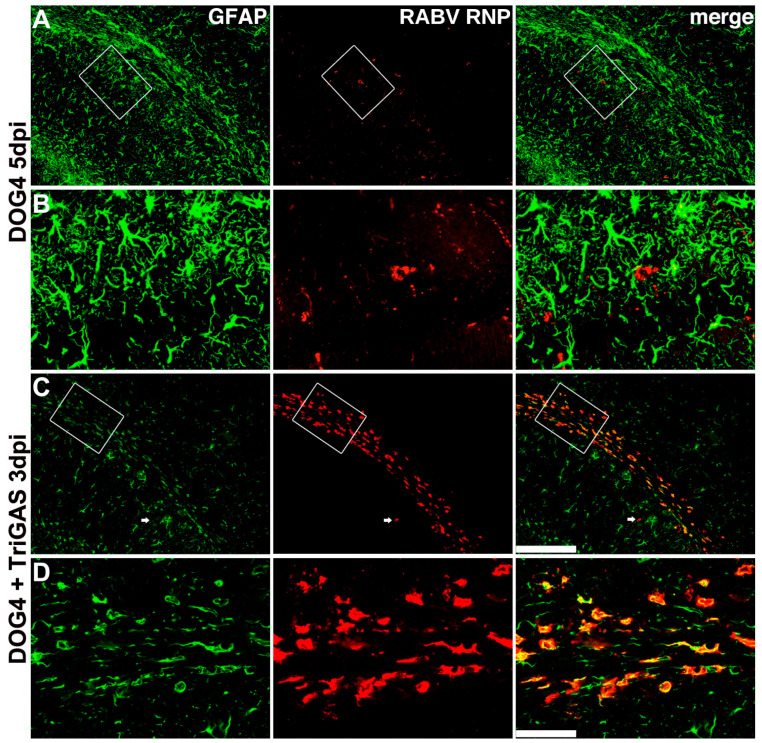
TriGAS infection progressed faster than DOG4 infection in co-infected brains. Representative double immunofluorescent staining (two to eight sections from one to two animals per experimental group) for RABV ribonucleoprotein (RNP; red) and the astrocytic marker glial fibrillary acidic protein (GFAP; green) in brain sections 5 d.p.i. (i.c.) with DOG4 alone (**A**,**B**) and 3 d.p.i. (i.c.) with a combination of DOG4 and TriGAS (**C**,**D**). Panels (**A**,**C**) display the hippocampus (lower left) and neocortex (upper right), with the corpus callosum running diagonally (scale bar: 200 µm). Higher magnifications of infected cells, indicated by white rectangles in (**A**,**C**), are shown in panels (**B**) and (**D**), respectively (scale bar: 50 µm). The arrows in (**C**) highlight a single infected neuron in the hippocampus.

DOG4 infection was not detected in the first four days after inoculation (upper panels in Figure 6C,F,I,L). At 5 d.p.i., it predominantly infected neurons in the hippocampus (Figure 6O: upper panel) and diencephalon. Double immunofluorescence staining for GFAP and RABV RNP confirmed mutual exclusion of GFAP-positive astrocytes and viral antigen in DOG4-infected brains (Figure 7A,B). In co-infected mice, the viral distribution resembled that observed after TriGAS infection alone (Figure 6: left vs. middle columns; Figure 3E vs. Figure 7C,D), with only rare instances of neuronal infection (Figure 7C, arrow).

By 2 days p.i., TriGAS-infected astrocytes in the corpus callosum displayed cytopathic changes, retracted processes, and swelling. By 3 days p.i., infected astrocytes had largely disappeared, leaving behind small RNP-positive remnants. The absence of activated caspase-3 staining and complement C1q deposits ruled out apoptosis and complement-mediated lysis as clearance mechanisms.

One-step growth curves (Figure A3A,B) in primary astrocytes indicated support of viral genome replication (Figure A3H,I), transcription (Figure A3J,K), and virion release (Figure A3E,F). However, the infectious potential of progeny virus was reduced (Figure A2D–F; Figure A3C,D,G) and focus formation impaired (Figure A2D–I). In vitro, infection was not accompanied by necrosis or apoptosis.

In summary, our in vitro observations show that astrocytes were infected and produced viral progeny with reduced infectious potential. While the infection itself did not induce detectable cell death in vitro, in vivo, local immune activation coincided with the reduction in virus-positive astrocytes.

Immunohistochemical detection of CD3 was used to visualize the temporal emergence and spatial distribution of T cells over the first five days after i.c. inoculation with TriGAS or DOG4 alone or in combination. In DOG4-infected brains, only sparse vascular T cells were observed (Figure 6: right column, lower panels). In contrast, after co-infection, T cells accumulated around midline venous vessels above the corpus callosum within one day p.i. (Figure 6B; lower panels) and infiltrated the parenchyma by day 2 p.i. (Figure 6E; lower panels), similar to after TriGAS infection alone (Figure 6: left column, lower panels). Together, these observations show that during i.c. co-infection, the local CNS immune phenotype closely matched that observed after TriGAS infection alone and temporally coincided with the disappearance of detectable RABV antigen.

## 4. Discussion

We investigated the relationship between defined glycoprotein substitutions in recombinant RABV strains and infection patterns, immune readouts, and disease course in a murine i.c. infection model. The combined RABV G R333E and N194S substitutions were associated with an astrocyte-restricted infection pattern, amoeboid microglia morphology, immediate T-cell infiltration, and early endothelial SELP expression. The astrocyte activation marker nestin showed distinct spatial patterns across variants. After inoculation with SPBNGAK, nestin was detected within virus-positive astrocytes, whereas after inoculation with GAS variants, it was restricted to astrocytes surrounding infected cells. These patterns, rather than astrocyte infection per se, correlated with infection outcomes in our model. Infection with the astrocytotropic TriGAS coincided with early CNS immune activation and survival in a simultaneous i.c. co-infection challenge with a highly virulent RABV strain. Because our analyses were primarily qualitative, these findings do not establish mechanistic causality. However, they identify astrocyte-restricted infection as a relevant correlate of distinct CNS immune response patterns that warrant further investigation.

Neurotropism has long been considered a hallmark of RABV. Infection of other cell types, including astrocytes, has been reported, but with inconsistent associations with pathogenicity. Studies have attributed astrocyte infection to pathogenic street viruses, including dog- and bat-originated strains, rather than to lab-adapted strains [22,72,73,74,75,76]. In our model, astrocyte infection was observed after i.c. inoculation with lethal SPBNGAK and apathogenic GAS variants, but not with the highly pathogenic DOG4 strain or after lethal i.c. inoculation with the attenuated recombinant SPBN strain. In contrast to Itakura et al. [27], who reported astrocyte infection with R333E-bearing RABV, SPBNGA infections remained restricted to neurons in our study. Accordingly, within our strain set and i.c. model, astrocyte infection did not correlate with pathogenicity, strain origin, or the RABV G mutation R333E alone. Instead, substitutions at position 194 within RABV G (N194K or N194S) in an R333E background were associated with infection patterns that included astrocyte involvement.

RABV G mediates cell invasion [14] and interacts with cellular receptors [17,18,19,20]. Mutations in the glycoprotein may affect cell tropism and internalization. Mutations at position N194 have been shown to counteract the delayed internalization and limited cell-to-cell spread characteristic of apathogenic R333 mutants [37,77]. This is consistent with our observation that both aa194 variants were associated with higher viral loads than R333E alone. The altered distribution pattern and astrocyte-restricted infection observed after i.c. SPBNGAK and GAS inoculations suggest that substitutions at position 194 in an R333E background are associated with altered infection patterns, without permitting conclusions about the underlying molecular mechanisms. Several mechanisms may contribute. One possibility is altered receptor usage. N194 lies within a region of RABV G that shows sequence similarity to snake neurotoxins binding nicotinic acetylcholine receptors (nAChRs) [36]. In addition, astrocytes and neurons differ in receptor expression and accessibility. Substitutions at R333 and N194 may also affect post-entry processes, such as glycoprotein processing, virion assembly, or budding efficiency. These effects may occur in a cell type-dependent manner and influence productive infection independently of initial attachment. In addition, residue N194 is located in a region implicated in glycoprotein structure and glycosylation [78], raising the possibility that altered glycosylation or conformational dynamics contribute to astrocyte-restricted infection. Differential binding kinetics between mutant and wild-type virions to astrocytic versus neuronal membranes could further bias cellular infection patterns. These mechanistic possibilities remain speculative, as receptor usage, binding kinetics, glycosylation, and post-entry processes were not directly examined in the present study. Nevertheless, the reproducible astrocyte-restricted infection observed across animals argues against stochastic infection events. Identifying the molecular determinants associated with selective susceptibility of fibrous astrocytes of the corpus callosum [21] will require direct experimental testing. In this context, transcriptionally distinct “pro-viral astrocyte” subpopulations described in human astrocytes [79] may further contribute to permissiveness.

The route of inoculation was associated with differences in viral tropism. After i.c. inoculation, GAS variants showed astrocyte-restricted infection patterns and substantial viral antigen in the brain. In contrast, peripheral inoculation resulted in reduced cerebral infection that was restricted to neuronal cells. These findings are consistent with prior reports of route-dependent limitations in brain access for apathogenic strains [76], including TriGAS [57]. Astrocyte infection has been reported after i.c. but not intramuscular inoculation with rCVS-11 [72]. In vitro, GAS variants did not differ from SPBN in primary corpus callosum astrocyte cultures or neuroblastoma cells regarding replication, transcription, virion release or infectious potential of viral progeny. This is in line with RABV’s broad in vitro tropism [80,81]. Together, these observations suggest that in vivo tropism may reflect not only receptor specificity [17,18,19,20] but also cell type-specific innate responses [82]. These conclusions are limited to the inoculation routes and time points examined in the present study.

Engineering RABV G has been used to alter immune recognition and pathogenicity [83,84]. Residue R333, located in antigenic site III, is critical for immune recognition [33,85]. In our study, the R333E substitution was associated with differences in immune response induction and magnitude, while residue 194 correlated, in this specific experimental context, with differences in clearance and survival outcomes. In neuron-restricted SPBNGA infection, the combination of R333E with N194 coincided with local immune responses and viral clearance. Reduced neurovirulence and viral burden may have contributed to immune activation during SPBNGA infection. However, this alone does not explain the divergent outcomes seen with SPBNGAK and the GAS variants. Although viral antigen levels at 3 d.p.i. were comparable, GAS variants, unlike SPBNGAK, were not lethal after i.c. inoculation. The presence of SPBNGAK elicited local immune responses and parenchymal T-cell infiltration, but was not associated with survival, despite astrocyte infection. These findings contrast with reports linking astrocyte infection to attenuation through enhanced type I interferon responses [27,73,74]. This suggests that viral burden and reduced neurovirulence alone do not account for immune outcome. GAS variants were associated with early microglial transition to an amoeboid state and endothelial SELP expression that persisted for at least 20 days. In contrast, infection with SPBNGA provoked these features only at later time points. The mechanisms underlying persistent immune activation after viral clearance remain unresolved, and sustained neuroinflammation may have adverse consequences [86]. While non-cytolytic neuronal clearance can occur without severe pathology [87], altered neuronal function has been reported [88]. Peripheral inoculation with GAS variants resulted in delayed cerebral immune responses, consistent with route-dependent timing of RABV immunity [89]. Apathogenic GAS variants induce protective immunity after peripheral administration despite limited CNS invasion and conserved neurotropism [90,91,92,93]. However, our data suggest that the early local immune activation observed in this model may be restricted to direct CNS exposure. Immune activation and clearance were inferred from histological and temporal patterns rather than from direct functional immune assays.

SPBNGAK and GAS infections differed not in viral load or immune activation but in the cellular context of infection and the associated astrocyte response. Our data are consistent with two patterns of astrocyte involvement, distinguishable by nestin expression. In the presence of the RABV G mutation R333E, nestin expression was observed regardless of astrocytotropism; with N194K (SPBNGAK), nestin co-localized with viral antigen in astrocytes, whereas with N194S (GAS variants), nestin expression and astrocyte infection were mutually exclusive. These differences are associated with variation in astrocyte activation states and may relate to differences in the local immune environment, without implying a defined immunomodulatory mechanism. Nestin is a marker of astrocyte reactivity [94,95,96,97], and its expression around SPBNGA-infected neurons and within SPBNGAK-infected astrocytes is consistent with astrocyte activation in the presence of viral infection. Neurotropic infection alone, such as with SPBNGA, has also been reported to induce astrocyte activation [98], and has been observed with neuroinvasive viruses [21,99,100]. Astrocyte involvement in antiviral defense has been described in neurotropic viral infections, highlighting their broader role as innate immune modulators within the CNS. Reactive astrocytes have been reported to express interferon-stimulated genes [73] and participate in virus sensing [101], contributing to interferon-dependent antiviral responses [90,102]. Nestin expression in the present study was used as a marker of astrocyte reactivity and does not provide direct information on functional immune signaling.

Abortive astrocyte infection [73] has been reported even when neurons are the preferred host cells [21,103]. Astrocytes are important sources of IFN-β in such contexts [104]. We found no astrocyte infection by SPBN or DOG4. However, our data suggest that SPBNGAK infection resembles the infection pattern of a pathogenic virus, as described by Potratz et al. [72]: astrocytes became activated by their infection, as evidenced by nestin expression. This may have contributed to an innate immune response that restricted viral replication in astrocytes but not in neurons. Nonetheless, the neuronal load disrupted the circuits and was lethal. Our in vitro data from primary callosal astrocytes suggest that “abortive” infection may reflect limited cell-to-cell spread rather than failed viral replication, consistent with restricted RABV spread from astrocytes [74] and absent shedding of infectious virus by 2GAS [105,106].

In contrast, GAS-infected astrocytes lacked nestin expression in vivo, suggesting differences in astrocyte activation states. Based on published reports of enhanced postexposure protection by an IFN-*γ*-expressing GAS variant [90] compared to 2GAS in mixed i.c. infections with DOG4 in mice [107], one possible interpretation is that GAS variants favor lytic loss of infected astrocytes rather than an abortive, high type-I-IFN state, leaving cells unresponsive to IFN-γ from activated glia. Hypothetically, elimination of infected astrocytes could be associated with the release of danger-associated molecular patterns (DAMPs) that promote the transition of microglia to an amoeboid, phagocytic state [108], potentially contributing to sustained immunoactivity. However, astrocyte fate, cytokine profiles, and cell death pathways were not directly assessed in the present study. Neuronal apoptosis with attenuated/apathogenic RABV has been well documented [109,110,111,112], especially with high G expression [12,113]. However, we observed no caspase-3 activation in vivo, and astrocytes survived infection with all viruses tested in vitro, showing no signs of apoptosis or necrosis. Both SPBNGAS and TriGAS were effectively cleared from the CNS, despite differences in RABV G expression. Elucidating astrocyte death pathways will be important for understanding sustained immune activation after viral clearance.

Effective therapies are lacking once RABV reaches the CNS; interferon, tribavirin, and passive antibodies fail to clear established infections [1,114,115]. Neuroprotective coma, nonspecific critical care, and hypothermia have not improved outcomes in human rabies [3]. CNS-localized immune activation [116] has been associated with viral clearance in experimental models, including innate signaling [40,73,102,107] and local antibody production [40,117,118]. High VNA titers alone [117] require concurrent BBB opening to be effective [41,42,119]. Although artificial, i.c. inoculation models a defined time point of brain entry and permits controlled analysis of interventions. In this model, the apathogenic astrocytotropic TriGAS infection differed from the pathogenic neurotropic DOG4 infection at early time points. Viral antigen was detected in astrocytes after TriGAS by day 1, whereas DOG4-infected neurons were detected only from day 5, consistent with delayed antigen exposure reported for DOG4 [69].

Detection of viral antigens in astrocytes after TriGAS inoculation, alone or in co-infection, was associated with early CNS immune activation and T-cell recruitment. In previous co-infection experiments, survivorship and viral burden were influenced by the relative abundance of apathogenic versus pathogenic RABV G expression [45]. TriGAS carries 3 copies of the G gene, whereas DOG4 suppresses G expression [69]. This imbalance may contribute to earlier immune activation in this model [57]. These observations are consistent with the possibility that timely innate immune responses can limit pathogenic RABV spread before extensive neuronal involvement with lethal consequences occurs. However, therapeutic efficacy, safety, and generalizability cannot be inferred from the present study. Because immune activation after TriGAS inoculation preceded the reported onset of VNA production, protection in this model may involve early chemokine-mediated CNS immune response [89,93,120]. Whether such responses can be harnessed therapeutically, and under what conditions, will require direct functional testing in clinically relevant models.

## 5. Limitations

Several limitations of this study should be acknowledged. First, our analyses were primarily qualitative and focused on characterizing viral tropism patterns, cellular distribution, and temporal immune readouts rather than providing quantitative measures of infection efficiency or immune response magnitude. Differences in pathogenicity and survival between viral strains further precluded direct quantitative comparisons across conditions. Quantitative approaches based on cell dissociation, such as flow cytometry, were evaluated with brain tissue from TgN(hGFAP-EGFP)GFEA mice but were unsuitable for this study. Fibrous astrocytes were difficult to recover intact, and dissociation procedures introduced strong biases against this cell population. Additionally, these procedures eliminated spatial information that is essential for defining CNS cell tropism in vivo.

Second, we did not include recombinant viruses carrying isolated substitutions at position 194 in the absence of the R333E mutation. This limits our ability to assess the independent contribution of residue 194 to viral tropism, pathogenicity, and immune responses outside the R333E background examined here.

Third, all conclusions are based on associations observed in a defined experimental setting, namely, i.c. inoculation in female Swiss Webster mice. The findings may therefore not be generalizable to other routes of infection, host species, or natural transmission conditions.

Fourth, although we observed reproducible phenotypic patterns associated with specific glycoprotein substitutions, the molecular and cellular mechanisms underlying these associations were not directly investigated. Receptor usage, intracellular signaling pathways, astrocyte fate, and immune effector mechanisms remain unresolved and will require targeted functional studies.

Finally, while astrocyte-restricted infection was associated with distinct immune response patterns in this model, the present study does not address therapeutic efficacy, safety, or biodistribution. Any consideration of translational applications will require extensive studies beyond the scope of the current work.

## 6. Conclusions

Attenuated live rabies virus vaccines have been explored as potential tools to induce immune responses within the CNS when classical PEP protocols are no longer effective. Experimental studies have shown that intrathecal or i.c. administration of live RABV can elicit local immune responses and, under defined conditions, promote viral clearance [116,120,121]. In the present study, the apathogenic RABV variant TriGAS was associated with an astrocyte-restricted infection pattern, early CNS immune activation, and survival in a murine i.c. co-infection model with a highly virulent RABV strain. These outcomes occurred in the context of defined amino acid substitutions within RABV G and were inferred from histological and temporal patterns rather than from direct functional immune assays. Importantly, the present data do not establish causal mechanisms linking astrocyte infection, immune activation, and protection. Comparison with SPBNGAK further indicates that astrocyte involvement alone is insufficient to predict outcome, as mixed neuronal-astrocytic infection was associated with immune activation but not survival despite comparable early viral antigen levels. These observations argue against viral burden as the sole determinant of immune outcome. They suggest that the cellular context and activation state of infected astrocytes may modulate CNS immune responses. However, this interpretation remains associative and requires direct experimental validation. The present study does not address key aspects relevant for therapeutic development, including long-term safety, viral persistence, biodistribution beyond the brain, and functional consequences of sustained cerebral immune activation. Astrocytes play central roles in CNS homeostasis, and their infection may have context-dependent effects that warrant careful evaluation. Consequently, any consideration of RABV variants with stable astrocyte tropism for therapeutic application requires comprehensive safety and mechanistic studies beyond neurovirulence.

Future work should define the fate of infected astrocytes and the molecular pathways associated with immune activation and clearance by, for example, comparative single-cell-transcriptomic analyses and functional immune profiling. While astrocyte-restricted RABV infection was associated with distinct immune response patterns and protection from lethal challenge in this experimental model, its translational relevance remains speculative and cannot be inferred without further extensive investigation.

## Data Availability

The raw data supporting the conclusions of this article will be made available by the authors upon request.

## Abbreviations

The following abbreviations are used in this manuscript:

2GAS	Recombinant SPBN-based RABV variant carrying two copies of the GAS glycoprotein gene
Acc	Primary astrocytes from the murine corpus callosum
AIF-1	Allograft inflammatory factor 1
AI	Artificial intelligence
ANOVA	Analysis of variance
AraC	Cytosine arabinoside
AUC	Area under the curve
BBB	Blood–brain barrier
BSR	Baby hamster kidney (BSR) cell line expressing T7 polymerase
C1q	Complement component 1q
CCL4	Chemokine (C-C motif) ligand 4
CD3	Cluster of differentiation 3 (T-cell marker)
cc	Corpus callosum
CNS	Central nervous system
Ct	Threshold cycle
Cx	Neocortex
DAMPs	Danger-associated molecular patterns
DAPI	4′,6-diamidino-2-phenylindole
DIV	Days in vitro
DMEM	Dulbecco’s Modified Eagle Medium
DNA	Deoxyribonucleic acid
DNase I	Deoxyribonuclease I
DOG4	Highly pathogenic dog-originated rabies virus strain DOG4
DRV4	Dog rabies virus isolate 4 (original designation of DOG4)
d.p.i.	Days post infection
FAD	Fluorescent antibody dilution buffer
FBS	Fetal bovine serum
FFU	Focus-forming unit
G	Glycoprotein
GAS	RABV G gene carrying the R333E pathogenicity determinant and N194S substitution
GFAP	Glial fibrillary acidic protein
Hpc	Hippocampus
h.p.i.	Hours post infection
IC-LD50	50% intracerebral lethal dose
IFN(-β, -γ)	Interferon (beta, gamma)
IL-1β	Interleukin-1 beta
i.c.	Intracerebral
i.n.	Intranasal
L	RNA-dependent RNA polymerase (large protein)
LOEWE	Initiative for Excellence in Science and Technology of the state of Hessen
M	Matrix protein
m.o.i.	Multiplicity of infection
mGluR2	Metabotropic glutamate receptor 2
NA	Neuroblastoma cell line of A/J mouse origin
nAChR	Nicotinic acetylcholine receptor
NCAM	Neural cell adhesion molecule
N	Nucleoprotein
P	Phosphoprotein
PBS	Phosphate-buffered saline
PCR	Polymerase chain reaction
PEP	Postexposure prophylaxis
qPCR	Quantitative polymerase chain reaction
RABV	Rabies virus
RABVG	Rabies virus genome
RNA	Ribonucleic acid
RNA-seq	RNA sequencing
RNP	Ribonucleoprotein
RPMI	Roswell Park Memorial Institute medium
RT	Reverse transcription
RT-PCR	Reverse transcription polymerase chain reaction
RT-qPCR	Reverse transcription–quantitative polymerase chain reaction
SAD B19	Street Alabama Dufferin B19 rabies virus strain
Sbt	Sorbitol
SELP	P-selectin (endothelial activation molecule)
SEM	Standard error of the mean
SFM	Serum-free medium (OptiPro SFM)
SPBN	Recombinant derivative of SAD B19 with pseudogene deleted and modified G locus
SPBNGA	SPBN variant carrying R333E mutation in G (“GA”)
SPBNGAK	SPBN variant carrying N194K and R333E mutations in G (“GAK”)
SPBNGAS	SPBN variant carrying N194S and R333E mutations in G (“GAS”)
SVZ	Subventricular zone
VNAs	Virus-neutralizing antibodies
WHO	World Health Organization

## Appendix A

**Table A1:** Oligonucleotides for RT-qPCR.

Name	Nucleotide Sequence (5′-3′)	Target, Sense
MP079	ACACCCCTACAATGGATGC	RABV genome, forward
MP080	GGGTTATACAGGGCTTTTTCA	RABV genome, reverse
MP081	ACAGGAGGCATGGAACTGAC	RABV N mRNA, forward
MP082	TGTTTTGCCCGGATATTTTG	RABV N mRNA, reverse

Abbreviation: RT-qPCR, reverse transcription-quantitative polymerase chain reaction.

**Table A2:** Parameters for the quantification of RABV genomes (RABVG) and RABV N mRNA.

Parameter	RABVG (MP079/MP080)	RABV N mRNA (MP081/MP082)
Standard line equation	y=−3.3202 x+36.849	y=−3.3219 x+36.151
PCR efficiency	100%	100%
*R* ^2^	0.99981	0.99953
Limit of quantification	32.12 Ct	29.36 Ct
Limit of detection	10^2^ amplicons	10^2^ amplicons
Linear range	10^2^–10^7^ amplicons	10^2^–10^7^ amplicons

Abbreviation: Ct, threshold cycle.
